# An Unusual Case of Cutaneous *Nocardia cyriacigeorgica* Infection Assuming the Appearance of Shingles

**DOI:** 10.1155/crdi/6056961

**Published:** 2026-03-10

**Authors:** Jett Ramir, Denise Marie A. Francisco

**Affiliations:** ^1^ University of Illinois College of Medicine at Peoria, 1 Illini Drive, Peoria, Illinois, 61605, USA, uic.edu; ^2^ Department of Internal Medicine–Infectious Diseases, University of Illinois College of Medicine at Peoria, 1 Illini Drive, Peoria, Illinois, 61605, USA, uic.edu

## Abstract

*Nocardia* is an opportunistic organism that commonly infects the lungs of the immunocompromised and can cause disseminated infections. Diagnosis of *Nocardia cyriacigeorgica* is through culture and susceptibilities, aided by 16S rRNA sequencing. We report a case of a shingles‐like rash eruption due to *Nocardia cyriacigeorgica* in a 69‐year‐old patient with a history of bilateral lung transplant.

## 1. Introduction

*Nocardia* is a Gram‐positive, aerobic, partially acid‐fast, beaded, branching rod that is found in soil and aquatic habitats [1–3]. It is characterized by branching structures that also stain partially acid‐fast. There are over 50 different species within the *Nocardia* genus. It inhabits our environment in soil, water sources, and decaying plant matter [3]. Humans encounter *Nocardia* through inhalation most commonly but can also be exposed via open wounds. The importance of the species is that *Nocardia* is an opportunistic bacterial pathogen, making it particularly dangerous to people who have a compromised immune system. These immunocompromised individuals who are infected by *Nocardia* usually manifest as pulmonary infections that can disseminate throughout the body in severe cases [4].

## 2. Case Presentation

**FIGURE 1:**
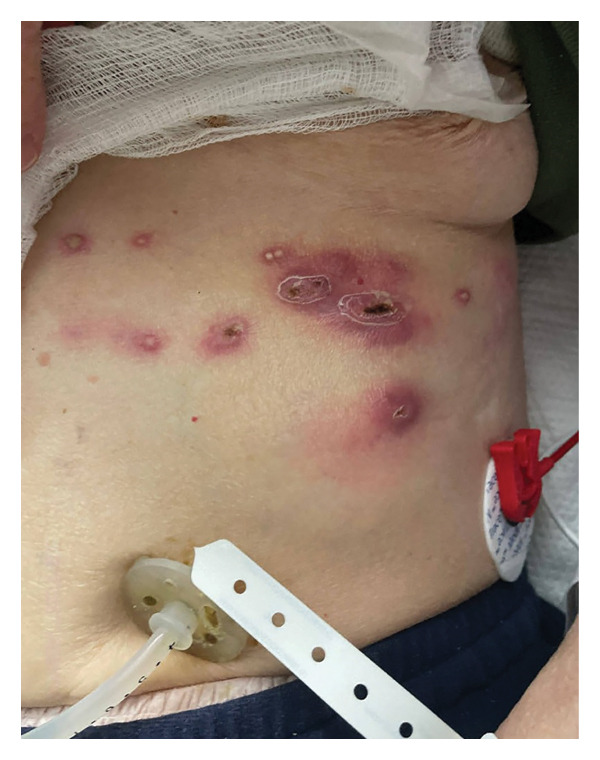
Initial presentation in the emergency department, status post incision and drainage, with a vesiculopustular rash overlying an erythematous base, with a few satellite pustular lesions spanning the left seventh thoracic dermatome.

**FIGURE 2:**
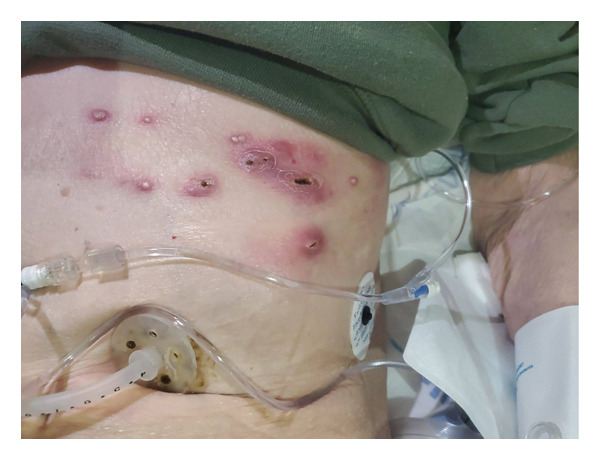
Photo documented by the infectious diseases team later in the emergency department, with residual crusting and scale noted.

A 69‐year‐old woman with idiopathic pulmonary fibrosis, status post bilateral lung transplantation, presented to the ED in July 2024 with extreme fatigue and a new rash that started three days prior. On physical examination, a chronically ill, pale‐appearing woman with tachycardia was noted; otherwise, she was hemodynamically stable and without cardiopulmonary findings. Examination of the skin showed a vesiculopustular rash overlying an erythematous base, with a few satellite lesions spanning the left seventh thoracic dermatome (Figures 1 and 2). Residual crusting and scale were seen where incision and drainage were performed at an outpatient center the day prior. There was warmth and exquisite tenderness to palpation of the rash and the left side of the body. On the right forearm, a tender, indurated nodule with no overlying erythema or warmth was present.

Since the lung transplantation operation in 2015, she had been treated with immunosuppressants including mycophenolate, tacrolimus, and prednisone. Her family noted that despite being immunocompromised, she had been remarkably free of complications for the past 9 years. That was until 2 months prior to presentation, when she experienced a presumably unrelated cerebrovascular accident. While at the rehabilitation facility, she had failed to maintain her weight, and a percutaneous endoscopic gastrostomy (PEG) tube was placed for enteral nutrition. One month after the cerebrovascular accident, she presented to the emergency department for a ground‐level fall that occurred in the rehabilitation facility, which resulted in a closed hip fracture.

After finally being discharged from the skilled nursing facility, she presented to her primary care physician with the rash in question. That is when the incision and drainage were performed, and bodily fluid from the lesion was cultured on Gram stain: *Staphylococcus epidermidis* was plated, which was thought to be a contaminant.

The next day, the patient’s husband noticed that there were more pustules appearing on the patient’s chest, and that she was no longer acting like herself. Thus, the patient and family presented to the emergency department. The infectious diseases team was consulted by the emergency department physicians because an infectious etiology was on top of the differential, and the patient’s medical history included multiple immunosuppressive agents. Infectious diseases initially recommended piperacillin–tazobactam and vancomycin empirically for broad‐spectrum bacterial coverage. Because the rash was dermatomal in distribution and the patient had a history significant for varicella‐zoster virus infection, shingles infection was also considered; valacyclovir was also added to the regimen.

On the third day of hospitalization, the patient’s physical and mental status had deteriorated despite her anti‐infective regimen. Ultrasound of the extremities was suggestive of an abscess in the right forearm. Computed tomography of the chest revealed small cavitated nodules in the right lower lobe and subcutaneous nodules along the chest. The nodule in the lung, along with the cutaneous abscess/mass and the patient’s immunocompromised state, raised suspicions for fungal infection.

Later that day, the original culture from the primary care physician’s office—which was collected 4 days prior—demonstrated Gram‐positive, branching filamentous rods consistent with *Nocardia* species that were pan‐susceptible to antibiotics. With the etiology of the cutaneous rash now known, we planned to move forward with first‐line treatment for *Nocardia*: trimethoprim–sulfamethoxazole. However, the patient had experienced adverse reactions to sulfa antibiotic drugs in the past. Second‐line treatment for *Nocardia* infection includes a multidrug regimen, thus imipenem–cisplatin and linezolid were started. Fungal workup in the blood had then become positive for *Cryptococcus* Ag, and a spinal tap confirmed *Cryptococcus* in the central nervous system. At this time, her anti‐infective regimen was composed of imipenem–cisplatin, linezolid, amphotericin B, and flucytosine.

The woman’s health status continued to deteriorate despite the continued effort. Subsequent testing revealed BK virus antigen in the urine. Positron emission tomography–computed tomography (PET–CT) scan elicited numerous hypermetabolic soft tissue masses involving the body wall and soft tissues of the right upper and lower extremities, which likely correlated with a disseminated infection. After difficult conversations with the patient and her family, they ultimately decided to discontinue efforts at aggressive treatment of the numerous infections. Her care transitioned to comfort measures only. A few days later, while in the comfort of her home, she passed away.

## 3. Discussion

### 3.1. Microbiology

*Nocardia* is a Gram‐positive, aerobic, partially acid‐fast, beaded, branching rod that is found in soil and aquatic habitats [1–3]. It can be grown on most routine media; however, the use of specific media—buffered charcoal yeast extract—may increase the sensitivity [5]. Since *Nocardia* can take days to weeks to identify itself on media, there are new methods by which the bacteria can be identified, which are now preferred. The gold standard for diagnosing *Nocardia* infection is through identification and amplification of the 16S rRNA sequence, via culture‐dependent methods [5]. *Nocardia cyriacigeorgica* is a relatively new species within the genus *Nocardia* that was differentiated in 2001 [1]. The *N. cyriacigeorgica* complex contains about 295 virulence factors, one of them being the *mce* family gene, which is essential in the bacterium’s ability to infect and inhabit mammalian epithelial cells [3]. It can infect both immunocompetent and immunocompromised patients [2]. The clinical significance of the *N. cyriacigeorgica* is that the species and genus are opportunistic pathogens that can cause serious infections in those who are inoculated with it, generally manifesting as lung, skin, or central nervous system infections. Other notable species within the *Nocardia* genus include *N. asteroides*, *N. nova*, *N. transvalensis*, and *N. farcinica.* [1].

### 3.2. Cases Reported

A search on PubMed of “*Nocardia cyriacigeorgica*” with the filters of “human,” “case reports,” and indexed for MEDLINE yielded 57 results. Of those 57 cases, only eight of the reports involved cutaneous lesions, as seen in Table 1. Three out of the eight cases involved immunocompromised patients [9–11]. Four cases involved cutaneous abscesses [6–10], two cases presented as cellulitis [11, 12], one case involved an oral abscess with draining sinuses to the chin [13], and one case was a pulmonary abscess with a cutaneous nodule [9].

**TABLE 1:** Table showing reported cases of *Nocardia cyriacigeorgica* causing various types of skin infections.

Reference	Age, y	Sex	Comorbidities	Infection type	Antimicrobial therapy
Alp, et al. [6]	32	M	Histiocytosis X	Pulmonary and cutaneous abscesses	Ceftriaxone (4 g/day) and amikacin (1 g/day)
Cargill, et al. [7]	85	F	COPD, polymyalgia rheumatica	Endocarditis and abscesses	Sensitivities: tetracycline, erythromycin, gentamicin, and imipenem. Resistance: penicillin, ciprofloxacin, teicoplanin, vancomycin, rifampicin, and fusidic acid.
Mahajan [8]	70	M	COPD, pulmonary silicosis	Cerebral and subcutaneous abscesses	Meropenem, linezolid, trimethoprim–Sulfamethoxazole
Chavez, et al. [9]	58	M	N/A	Lung abscess and skin nodule	
Özgenç, et al. [10]	45	F	None	Empyema and cutaneous abscesses	Imipenem, amikacin, and doxycycline for 45 days. Linezolid and doxycycline for 3 months. Trimethoprim–sulfamethoxazole for 16 months
Murata, et al. [11]	71	M	Type II diabetes mellitus	Cellulitis	Minocycline (100 mg/day) for 4 months
Rath, et al. [12]	32	M	N/A	Periocular/orbital cellulitis	Amikacin (1 g/day) and amoxicillin–clavulanate (625 mg BID) for 2 weeks. Amikacin (1 g/day) alone for 2 months
Rathore, et al. [13]	45	M	N/A	Oral abscess with cutaneous sinuses	Co‐trimoxazole for 4 months

*Note:* The table has characteristics of the affected patients including their age, sex, comorbid conditions, infection type, and antimicrobial therapy used to treat the *Nocardia cyriacigeorgica* infection. M, male; F, female; g, grams; mg, milligrams; BID, twice per day.

Abbreviation: COPD, chronic obstructive pulmonary disease.

### 3.3. Clinical Presentation

*Nocardia* infection classically presents as a pulmonary infection in an immunocompromised patient that can result in disseminated infection [3]. Common immunosuppressed populations that *Nocardia* can infect include patients with solid organ/hematopoietic stem cell transplant, chemotherapy‐induced immunosuppression, or autoimmune/inflammatory conditions treated with corticosteroid/immunosuppressive agents [2]. Gram stain will elucidate the species; however, with the increased incubation time of *Nocardia*, other methods like 16S rRNA sequencing are preferred. *Nocardia* species are known to mimic other infections including sporotrichosis, atypical mycobacteria, leishmaniasis, and tuberculosis [14, 15]. Cutaneous manifestations of *Nocardia* tend to follow three modes: mycetoma/lymphocutaneous infection, superficial skin infections, and dissemination with secondary skin involvement [14]. The incidence of skin involvement after dissemination from the primary lung infection is only seen in 10% of infections [4].

Our patient presented with an extremely painful, vesiculopustular rash extending along the seventh thoracic dermatome. Because of her history of chicken pox as an adolescent, a shingles eruption was forefront in the differential. However, with the abnormal mental status of the patient, further investigation was necessary. Incision and drainage of the lesions with plating of the fluid yielded the identification of *Nocardia* after 4 days. Although our patient elected not to undergo further diagnostics, we ascertain that this case of *N. cyriacigeorgica* infection was likely due to a primary pulmonary infection with dissemination and secondary involvement of the skin.

### 3.4. Management

Treatment for nocardiosis depends on the presentation and the severity of the infection. Historically, trimethoprim–sulfamethoxazole has been the gold standard in treating *Nocardia*. However, a multidrug regimen has been proposed for infections that are more complex and severe. A systematic review of multidrug regimens used for the treatment of *Nocardia* was performed [5]. For primary skin infections, Margalit, et al. [5] recommend trimethoprim–sulfamethoxazole for three to 6 months as initial treatment with linezolid as a possible alternative. For isolated pulmonary infections, trimethoprim–sulfamethoxazole ± imipenem, amikacin, ceftriaxone, or cefotaxime for 6 months is recommended. Alternatives include linezolid ± imipenem, ceftriaxone, or cefotaxime. For CNS involvement, the study recommends trimethoprim–sulfamethoxazole and imipenem ± amikacin for nine to 12 months. Alternatives include linezolid with imipenem. Our patient could not receive trimethoprim–sulfamethoxazole as she had a history of allergic reaction to sulfa antibiotics. Thus, our antibiotic regimen included linezolid and imipenem. The risks and benefits of long‐term treatment for the patient’s serious infection were weighed with her and her family. Ultimately, they decided that comfort measures were preferred.

## 4. Conclusion

*N. cyriacigeorgica* is an emerging pathogen within the United States. It is an opportunistic infection that commonly inoculates patients through inhalation of environmental particulates or through direct invasion of the skin through open wounds. Many cases are localized infections, but in severe cases, the organism can disseminate throughout the body with penetration to the central nervous system. Cutaneous nocardiosis is a less common manifestation of the organism that is usually seen in *immunocompetent* patients. Diagnosing *Nocardia* infection through classical methods is difficult when urgent action is necessary, as the organism can take days to weeks to grow on cultured media. Treatment for simple infections is with trimethoprim–sulfamethoxazole when indicated, but multidrug regimens (linezolid, imipenem, amikacin, and ceftriaxone) can be useful when treating severe, disseminated infections. When caring for the health of the immunocompromised, it is important to maintain a broad differential that includes *N. cyriacigeorgica*.

## Funding

No funding was received for this manuscript.

## Ethics Statement

Verbal informed consent was obtained from the family of the patient for publication of this case report and accompanying images. The patient’s personal information was deidentified to ensure confidentiality. IRB review was not required.

## Conflicts of Interest

The authors declare no conflicts of interest.

## Data Availability

Data sharing is not applicable to this article as no datasets were generated or analyzed during the current study.
